# Cross-cultural adaptation and psychometric properties of the Kessler Distress Scale (K10): an application of the rating scale model

**DOI:** 10.1186/s41155-021-00186-9

**Published:** 2021-07-19

**Authors:** Evandro Morais Peixoto, Daniela Sacramento Zanini, Josemberg Moura de Andrade

**Affiliations:** 1 University of São Francisco USF, 105 Waldemar César da Silveira St, Jardim Cura D’ars, Campinas, SP 13045-510 Brazil; 2 Pontifical Catholic University of Goiás PUC-Goiáis, Praça universitária s/n, av. Universitária, Goiânia, GO 74605-220 Brazil; 3 University of Brasília UNB, Campus Universitário Darcy Ribeiro, ICC Sul, Asa Norte, Brasília, DF 70910-900 Brazil

**Keywords:** Psychological distress, Anxiety, Depression, Validity, Reliability

## Abstract

**Abstract:**

**Background:**

The Kessler Distress Scale (K10) is a self-report scale for the assessment of non-specific psychological distress in the general and clinical population. Because of its ease of application and good psychometric properties, the K10 has been adapted to several cultures. The present study seeks to adapt the K10 to Brazilian Portuguese and estimate its validity evidence and reliability.

**Methods:**

A total of 1914 individuals from the general population participated in the study (age = 34.88, SD = 13.61, 77.7% female). The adjustment indices were compared among three different measurement models proposed for the K10 through confirmatory factor analysis (CFA). The items’ properties were analyzed by Andrich’s Rating Scale Model (RSM). Furthermore, evidence based on relations to other variables (depression, stress, anxiety, positive and negative affects, and satisfaction with life) was estimated.

**Results:**

CFA indicated the adequacy of the bifactor model (CFI= 0.985; TLI= 0.973; SMR= 0.019; RMSEA= 0.050), composed of two specific factors (depression and anxiety) and one general factor (psychological distress), corresponding to the theoretical hypothesis. Additionally, it was observed multiple-group invariance by gender and age range. The RSM provided an understanding of the organization of the continuum represented by the psychological distress construct (items difficulty), which varied from −0.89 to 1.00; good adjustment indexes; infit between 0.67 and 1.32; outfit between 0.68 and 1.34; and desirable reliability, α= 0.87. Lastly, theoretically coherent associations with the external variables were observed.

**Conclusions:**

It is concluded that the Brazilian version of the K10 is a suitable measure of psychological distress for the Brazilian population.

## Introduction

Developed in 2001 for Australia’s National Survey of Mental Health and Wellbeing (Australian Bureau of Statistics, 2001; Kessler et al., 2002; Pereira et al., 2019), the Kessler Distress Scale (K10) is a self-report scale for the assessment of non-specific psychological distress in the general and clinical population. Available in its original ten-item version (K10) or a reduced six-item version (K6), both have been well accepted in the scientific community and in international studies (Andrews & Slade, 2001; Bessaha, 2015; Fassaert et al, 2009; Mewton et al, 2016).

Generally, psychological distress can be defined as non-specific psychological suffering despite sometimes being referred to and assessed as synonymous with depression, anxiety, or other psychological symptoms (Decat et al., 2009). It has been assessed by different instruments, the General Health Questionnaire (GHQ-12) and the Depression, Anxiety, and Stress Scale (DASS-21) (Goldberg & Williams, 1988; Lovibond & Lovibond, 1995a, 1995b; Pais-Ribeiro et al., 2004) being particularly worthy of note.

As it is a brief scale and easy to apply and to correct, the K10 has been used in psychopathological screening studies for anxiety and mood disorders. In an Australian sample, its efficiency in detecting mental disorders has been compared to other instruments such as the General Health Questionnaire (GHQ-12) via the ROC curve. The results show that K10 was significantly better than GHQ-12 in screening for anxiety and mood disorders. Moreover, K10 seems to demonstrate good sensitivity in identifying internalizing disorders in population studies, even in a population of older adults (aged 65 or over) (Anderson et al., 2013).

Findings relating to the K10’s psychometric properties in screening for psychological distress have been corroborated in other studies with populations in New Zealand, Japan, and Palestine (Browne et al., 2010; Easton et al., 2017; Furukawa et al., 2008), indicating that it is a good instrument for this purpose with good indices of validity evidence, sensitivity, and specificity (Anderson et al., 2013; Furukawa et al., 2003).

Indeed, due to its ease of application and good psychometric results, the K10 has been translated and adapted to diverse cultures in addition to those previously described. These include the German and Turkish cultures (Fassaert et al., 2016), Arabic/Palestinian cultures (Easton et al., 2017), Portuguese culture (Pereira et al., 2019), and Australian aboriginal culture (Bougie et al., 2016). In all of these, good validity evidence and reliability were found. More specifically, the internal consistency of the K10, measured via Cronbach’s alpha, varied between 0.88, in a population of Palestinian social workers (Easton et al., 2017), and 0.91, in Portuguese adults (Pereira et al., 2019).

In an adult population, Pereira et al., 2019 reported the K10’s sensitivity regarding sociodemographic variables, indicating that participants over the age of 40 and of lower social class, suffered higher rates of distress. In an Australian Aboriginal population, socioeconomic status also influenced responses to the K10. In addition to this variable, it was observed that women and individuals with a lower educational level had a higher level of distress. However, contrary to what happened in the Portuguese population, among the Aboriginal population, respondents over the age of 55 suffered significantly less distress than younger respondents (Bougie et al., 2016).

Although there is a degree of consensus in the literature regarding the psychometric qualities of the K10, such as the reliability and sensitivity in the identification of clinical groups, the same cannot be said in terms of their internal structure. The instrument was developed based on a single-factor structure, which was confirmed in later studies (Bougie et al., 2016). However, studies have recommended an adequacy of an internal structure made up of two factors, which group together items representing the symptoms of depression and anxiety (Easton et al., 2017). In another study using the K10, Brooks et al., 2006 indicated the adequacy of a first-order two-factor structure (depression and anxiety) with a second-order general factor (psychological distress).

Lastly, other studies seem to demonstrate that the internal structure may vary depending on the sample, with the one-dimensional structure best suited to community samples and the two-factor structure being best suited to clinical and psychiatric samples (Sunderland et al., 2012). Additionally, it can be observed that this disparity in terms of the understanding of internal structure occurs due to unawareness in the identification of a general factor which may represent psychological distress and specific factors (depression and anxiety). In order to overcome these problems, international studies have proposed a two-factor structure, the results of which have been more satisfactory than those presented by second-order models. In this case, the bifactor structure permits an assessment of latent structures that is, at the same time, one-dimensional and multidimensional, since part of the item variance is explained by a general factor (psychological distress) and another part explained by specific factors (depression and anxiety) (Reise, 2012).

It should be stressed that the bifactor structure has been used in the evaluation of the internal structure of other instruments that assess psychological distress, such as the Depression, Anxiety, and Stress Scale (DASS-21) (Cucina & Byle, 2017; (Peixoto et al: DASS-21: assessment of psychological distress by the Bifactor Model and item analysis, in press)); Self-Rating Depression Scale (SDS); and Symptom Checklist 90-Revised (SCL-90-R) (Chen, Lin, et al., 2020). Thus, this study considers the potential of this model to tackle the discussion concerning the instability of internal structure observed in the K10 studies. In this way, this study hopes to offer a contribution to the international literature, given that funding for research based on this data analysis methodology has not yet been raised for the evaluation of this instrument. In addition, there is a paucity of international studies that seek to assess the properties of the items that make up the K10, with the help of item response theory (IRT), for example.

Although the Kessler Distress Scale is a globally used instrument that is easy to apply and with recognized psychometric quality, no studies were found that could be adapted to the Brazilian context. In this regard, the present article aims to present validity evidence and reliability for the Brazilian version of the K10. The ten-item version was chosen because of the good results of screening for anxiety and depression disorders. In particular, the following stages of psychometric analysis were taken into consideration: (i) comparison of rival factor models described in the literature; (ii) invariance test of the measurement model according to the respondents’ sex and age group; (iii) estimate of item difficulty parameters, goodness-of-fit indices, and the association between the item properties and participants’ distress levels; and (iv) obtaining validity evidence based on the relationship with external variables.

## Method

### Participants

A total of 1914 individuals participated in this study (22.3% male, 77.7% female), aged between 14 and 86 (average = 34.88, SD = 13.61), from 24 Brazilian states plus the Federal District of Brasília. In regional terms, 56 (2.9%) were from the North, 571 (29.8%) from the Northeast, 537 (28.1%) from the Midwest, 587 (30.7%) from the Southeast, and 163 (8.5%) from the South.

As for educational level, 0.4% of participants reported having completed primary education, 0.8% did not complete high school, 5.1% completed high school, 26.6% did not complete higher education, 17.8% completed higher education, 9.0% started but did not complete a postgraduate course, and 40.2% completed postgraduate education.

### Procedure

The study followed the guidelines of the Brazilian National Health Council, considering Resolution No. 510/2016 which refers to research involving human beings. Participants were recruited through media advertisements and on social media networks (*Whatsapp*, *Facebook*, *Instagram*, etc.). Voluntary participation was confirmed by completing the free and informed consent form, guaranteeing anonymity, the possibility of withdrawal at any stage of the study without obligation, and allowing contact with researchers for more information about the study or if any complications arose from participation. Participants’ responses to the instruments were done virtually (online), via the *GoogleForms* software. All answers were recorded between April 4 and April 17, 2020, approximately 1 month after the start of the new coronavirus pandemic in Brazil.

#### Procedure and adaptation of the K10

For the K10 adaptation procedures, the stages advocated by the International Test Commission (2010) were followed, as well as those recommended by Borsa et al., 2012. Accordingly, two independent translations from English to Brazilian Portuguese were performed. Subsequently, agreement between the translations was assessed, and potential discrepancies were discussed among the evaluators in order to reach agreement. The version of the instrument resulting from this process was subjected for analysis by expert judges for an evaluation of semantics and instrument layout. Lastly, the Portuguese version of the instrument was back-translated into English. The final result was input to *GoogleForms* together with the study’s other instruments.

### Instruments

The data collection instruments used in this study are described below.

#### Kessler Psychological Distress Scale (K10)

The K10 scale for the assessment of psychological distress was translated and adapted for the Brazilian population. The instrument has 10 self-report items that assess the level of emotional distress over the previous 30 days. Responses to the items are given via a 5-point Likert scale (1=never to 5=all the time). Higher scores signify a higher level of psychological distress or psychic suffering.

#### Depression, Anxiety Stress Scale (DASS-21)

Developed by Lovibond & Lovibond, 1995a, 1995b, the Depression, Anxiety, and Stress scale (DASS-21) assesses these three constructs using three factors of the same name, composed of seven items each, totaling 21 items on the scale. The response scale is a 4-point Likert scale, ranging from 0 (did not apply at all) to 3 (applied a lot or most of the time). In Brazil, DASS-21 was adapted, and its psychometric properties were evaluated in several regions of the country with samples of adolescents (da Silva et al., 2016; Patias et al., 2016) with results that assure the three-factor structure and good reliability indicators between 0.83 and 0.96. The higher the score on each subscale, the more prevalent are the negative affective states evaluated. It is important to mention that this instrument has been successfully used among different populations during the COVID-19 outbreak (Chang et al., 2020; Chen, Chen, et al., 2020). Therefore, such evidence supports it as being a good external criterion for the K10.

#### Satisfaction with Life Scale (SWLS)

Developed by Diener et al., 1985, the *Satisfaction with Life Scale* (SWLS) assesses cognitive aspects of subjective well-being, being considered the gold standard for evaluation of the construct (Diener, 2000). The scale has five self-report items that assess the participants’ satisfaction with life via a 7-point Likert scale. The Brazilian version was adapted and validated by Zanon et al., 2013, their adaptation studies showing good validity evidence and reliability, as well as preliminary norms (Hutz et al., 2014).

### Sociodemographic and social isolation questionnaire

For this study, a questionnaire was developed that sought to assess sociodemographic data such as age, sex, marital status, and family income level. Also developed were questions regarding adherence to social isolation during the pandemic, the existence of individuals identified as being within the risk group and children in the family group in social isolation, level of activity during social isolation, whether a positive diagnosis of COVID-19 was received, etc.

### Data analysis

For the assessment of the internal structure of the Brazilian version of the K10, confirmatory factor analysis (CFA) procedures were used, testing rival models. Specifically, CFA was used to compare rival models described in the literature, namely, (i) one-dimensional model, (ii) model with two correlated factors, (iii) second-order model, and (iv) bifactor model. To this end, CFAs were performed using the *Maximum Likelihood Robust* (MLR) estimation method, and the following fit indices were employed to assess the adequacy of the models: Chi-square (χ^2^), comparative fit index (CFI), Tucker Lewis index (TLI), standardized root mean squared residual (SRMR), root mean square error of approximation (RMSEA), and Akaike Information Criterion (AIC). The values commonly used in the literature were considered adequate: nonsignificant χ^2^ χ^2^/df < 3, CFI and TLI > 0.95, SRMR< 0.08, and RMSEA< 0.06. The AIC was employed to compare different models and determine which one is the best fit for the data; the model with the lowest AIC can be considered the better model.

Still on the topic of the assessment of the instrument’s internal structure, indicators of invariance in the measurement model were found regarding sex (male and female) and age groups (up to 30 years, between 30 and 55 and over 55) via multigroup CFA. For an assessment of the indicators of reliability, Cronbach’s alpha and McDonald’s omega coefficients were used, with values above 0.70 used as a reference. The statistics software Factor 10.10.1 (Ferrando & Lorenzo-Seva, 2017) and MPLUS (Muthén & Muthén, 2017) were used.

For an assessment of the psychometric properties of the items, the *Rasch-Andrich Rating Scale Model* (Graduated Response Model) was used, which is part of the *Rasch* family of measurement models and which is an extension of the dichotomous *Rasch* model for a sequence of graduated responses. As it is a one-dimensional one-parameter model, an estimation was performed of the levels of difficulty of the items, goodness-of-fit indices (*Infit*/*Outfit*), and the *theta* levels of the participants (Linacre, 2015). For a better representation of the association between item difficulty and intensity levels in the construct presented by the participants that made up the sample, the item map procedure (Embretson & Reise, 2000) was employed. These analyses were performed using the *Winsteps* statistical software and the *Joint Maximum Likelihood* estimation method (Linacre, 2015).

Lastly, analysis of Pearson’s *r* correlation was performed for estimation of the association between the scores presented by participants in the K10 factors and other external variables used in the study, such as, depression, anxiety, stress, psychological distress, positive and negative affects, and satisfaction with life. Significance levels below 0.05 were assumed.

## Results

In agreement with the first specific objective of the study, the goodness-of-fit indices of the following K10 models were compared: (i) one-dimensional model, (ii) model with two correlated factors, (iii) model with a second-order factor, and finally (iv) bifactor model. Results are shown in Table 1.

**Table 1:** Confirmatory factor analysis goodness-of-fit indices for the different K10 models evaluated

Model	χ^**2**^	df	χ^**2**^(df)	CFI	TLI	SRMR	RMSEA	(CI 90%)	AIC
One-factor	572.114	35	16.34611	0.862	0.823	0.056	0.130	0.123–0.136	52.341.761
Two-factor	618.981	34	18.20532	0.941	0.922	0.038	0.095	0.088–0.101	51.628.734
Second order	525.326	33	15.91897	0.940	0.918	0.037	0.088	0.082–0.095	51.630.522
Bifactor	146.898	25	5.87592	0.985	0.973	0.019	0.050	0.043–0.059	51.199.738

Note: all models were significant *p* < 0.001

*χ*^*2*^ Chi-square, *df* degree of freedom, *CFI* comparative fit index, *TLI* Tucker Lewis index, *SRMR* standardized root mean squared residual, *RMSEA* root mean square error of approximation, *CI* confidence interval, *AIC* Akaike Information Criterion

As shown in Table 1, the results indicate inadequate goodness-of-fit indices for the one-factor model and adequate values for the models with two correlated factors. The model with a second-order factor showed results similar to the model with two correlated factors for the K10. However, the bifactor model, consisting of two specific factors (depression and anxiety) and one general factor (psychological distress) showed significantly better goodness-of-fit indices compared to the other models, being the only one with results classified as good. Furthermore, this model showed the lowest AIC value, which indicates that it should be preferred over the other models tested due to its fit to the available data and its parsimony. Other results of the KI0 model can be seen in Table 2.

**Table 2:** Confirmatory factor model estimated for the Brazilian version of the K10

Item N°	Specific factors		General factor
	Depression	Anxiety	Distress
k01	.280 (.022)		.614 (.017)
k04	.322 (.021)		.550 (.018)
k07	.549 (.019)		.707 (.014)
k08	.345 (.023)		.561 (.018)
k09	.547 (.018)		.722 (.014)
k10	.539 (.020)		.585 (.018)
K02		.082 (.032)	.870 (.011)
K03		.029 (.026)	.882 (.011)
K05		.337 (.054)	.732 (.016)
K06		.686 (.123)	.713 (.020)

Notes: factor loading (standard error)

As for the factorial loads shown by the items, it can be seen in Table 2 that all have higher general factors than the factorial loads shown for the specific factors, denoting a larger variance of the general factor in the proposed structure. Particular emphasis should be accorded to the fact that items 1, 2, and 3 do not show factorial loads greater than or equal to 0.30 for the specific factors. As for the model’s estimated reliability indicators, the following values were observed for Cronbach’s alpha and McDonald’s omega coefficients, respectively: depression factor equal to 0.891 and 0.887, anxiety factor 0.886 and 0.886, and general distress factor 0.923 and 0.922.

A multigroup factor analysis (MGFA) was then performed for the evaluation of the measurement model’s invariance between the groups formed as a function of sex and age group. Results are shown in Table 3.

**Table 3:** CFA multigroup goodness-of-fit indices for the K10 as a function of sex and age group

	χ ^**2**^ (df)	χ^**2**^/df	CFI	TLI	SRM	RMESA (CI 90%)
**Sex**						
Configural	148.030 (50)	2.961	0.988	0.978	0.019	0.045 (0.037–0.054)
Scalar	209.311 (67)	3.124	0.982	0.976	0.032	0.047 (0.040–0.054)
**Age group**						
Configural	314.581 (67)	4.695	0.964	0.952	0.042	0.066 (0.059–0.073)
Scalar	266.682 (74)	3.604	0.972	0.966	0.040	0.055 (0.048–0.063)

Note: all models were significant *p* < 0.001

*χ*^*2*^ Chi-square; *df* degree of freedom, *CFI* comparative fit index, *TLI* Tucker Lewis index, *SRMR* standardized root mean squared residual, *RMSEA* root mean square error of approximation, *CI* confidence interval, *AIC* Akaike Information Criterion

The results exhibited in Table 3 indicate the invariance of the configural and metric model between the groups established as a function of participants’ sex (male and female) and age group (under 30, between 30 and 55 and over 55). This signifies that the instrument does not generate a difference in response by virtue of the participants’ sex or age group.

Armed with the initial validity evidence based on the internal structure and reliability of the Brazilian version of the K10, the psychometric properties of the instrument’s items were assessed via the IRT’s Graduated Response Model. The results are shown in Table 4. The table presents item difficulty level, goodness-of-fit indices, item-*theta* correlation indices, and reliability indicators, as estimated by the model.

**Table 4:** Results of the estimation of item parameters in the K10 based on the IRT

Items	***b***	***Infit***	***Outfit***	***Corr***	Reliability
K3	1.00	0.88	0.80	0.72	0.87
K6	0.55	1.18	1.14	0.66	
K10	0.46	1.31	1.19	0.69	
K9	0.44	0.70	0.68	0.78	
K4	0.19	1.26	1.34	0.64	
K7	−0.07	0.71	0.70	0.79	
K8	−0.41	1.32	1.30	0.67	
K1	−0.46	1.12	1.15	0.68	
K5	−0.81	0.87	0.92	0.71	
K2	−0.89	0.67	0.69	0.76	
M	0.00	1.00	0.99		
SD	0.60	0.25	0.25		

NB: items are shown in decreasing order of difficulty;

*b* difficulty of the item, *Corr* item-theta correlation (trait intensity level estimated by the model)

As observed in Table 4, the item difficulty indices presented a mean value of 0.0 and a standard deviation of 0.60. This strategy of setting the mean at 0.0 corresponds to an anchoring procedure that enables identification of measurements and association between item parameters and participants’ characteristics (*theta* levels). Thus, it can be seen that the items with levels of difficulty ranging from −0.89 (item 2) to 1.00 (item 3) and, consequently, the item set that makes up the K10 cover a *theta* range equivalent to 1.89 *logts*. As far as the goodness-of-fit indices are concerned, the *infit* statistic ranged from 0.62 to 1.32, while *outfit* ranged from 0.69 to 1.34. All indices proved to be adequate.

**Fig. 1:**
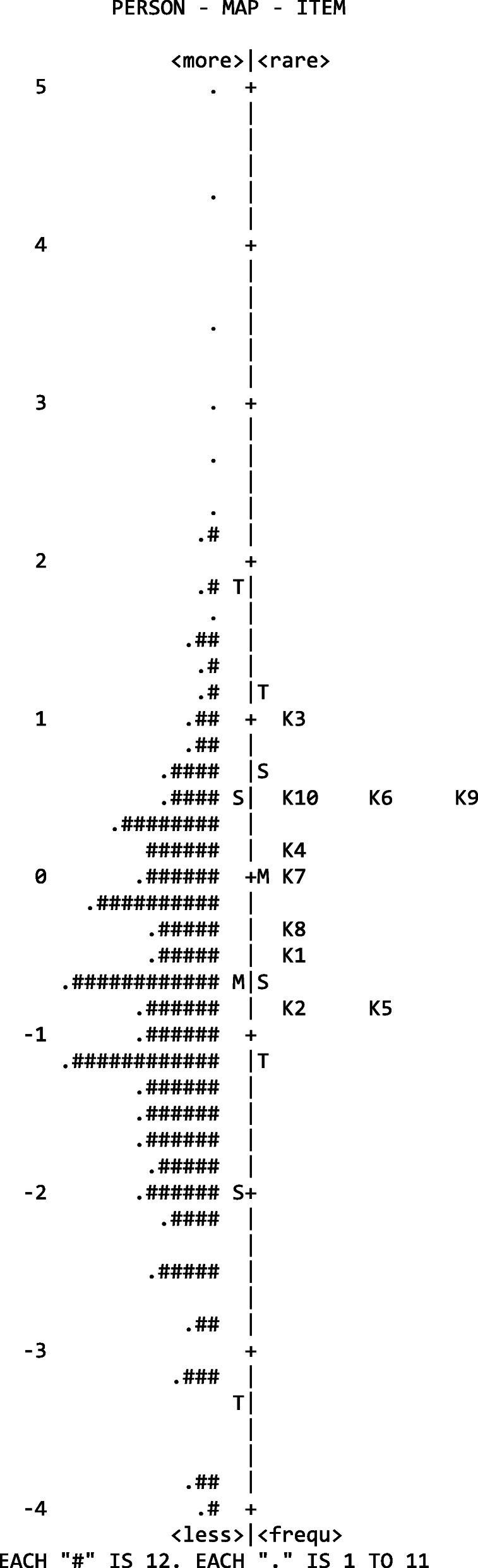
Person map item

Generally speaking, high correlations were observed between the item scores and *theta* levels shown by the participants (greater than or equal to 0.64). The reliability level for the set of items estimated by the model was shown to be very adequate, equal to 0.87. Lastly, an association was established between the level of item difficulty and the level of intensity (*theta*) shown by the participants. The results are displayed in Fig. 1.

On the horizontal axis in Fig. 1, reading from left to right, the representation of the latent trait can be observed first and foremost, which varies in a standardized metric between −4.0 and 5.0, with a mean value of 0.0 and a standard deviation of 1.0. The symbols “#” and “.” represent the number of participants allocated to the different latent trait levels; in this case, each “#” represents 12 people, and each “.” may represent up to 11 people. The symbols “M”, “S”, and “T” correspond to mean, one standard deviation, and two standard deviations, respectively. Lastly, the positioning of the items on the estimated latent trait continuum can be verified.

Thus, it can be seen that, although the items may cover a significant magnitude of the range of the latent trait level, these are more informative for the assessment of participants with a higher than average *theta* level. These results also suggest that the items are more difficult to confirm in participants with intensity levels lower than the average. This can be seen as an important strength of the instrument, considering that it was developed with the aim of being used to identify individuals with high levels of psychological distress.

The final stage of analysis was the estimation of validity evidence based on the relationship with external variables. For this purpose, correlations between K10 scores (anxiety, depression, and global psychological distress) and indicators of satisfaction with life and positive/negative affects were established and analyzed. Furthermore, the K10 scores were also correlated with the scores for depression, anxiety, stress, and global psychological distress from the DASS-21. Results are shown in Table 4, in which we may establish bivariate correlations between variables in studies.

Generally speaking, Table 5 exhibits correlation indices that corroborate the study’s hypothesis. Moderate and negative associations were observed between the anxiety factor and indicators of satisfaction with life and positive affects, as well as a high-magnitude positive correlation with negative affects. On the other hand, the depression factor and the general psychological distress factor showed moderate and negative correlations with indices of satisfaction with life and positive affects, as well as high-magnitude positive correlations with negative affects. These results support the hypothesis of convergent and divergent validity evidence of the K10 with measurements of satisfaction with life, positive affects, and negative affects.

**Table 5:** Correlation between the variables used in the study

	Anxiety K10	Depression K10	*Distress* K10	SWL	PA	NA	Depression DASS-21	Anxiety DASS-21	Stress DASS-21	Distress DASS21
Anxiety K10	1									
D-K10	.683^*^	1								
*Distress* K10	.875^*^	.952^*^	1							
SWL	−.293^*^	−.411^*^	−.396^*^	1						
PA	−.253^*^	−.454^*^	−.408^*^	.400^*^	1					
NA	.721^*^	.742^*^	.796^*^	−.336^*^	−.401^*^	1				
Depression DASS-21	.566^*^	.811^*^	.777^*^	−.443^*^	−.490^*^	.691^*^	1			
Anxiety DASS-21	.641^*^	.661^*^	.709^*^	−.271^*^	−.268^*^	.655^*^	.688^*^	1		
Stress DASS-21	.762^*^	.702^*^	.787^*^	−.295^*^	−.345^*^	.779^*^	.722^*^	.766^*^	1	
Distress DASS21	.725^*^	.804^*^	.840^*^	−.372^*^	−.407^*^	.784^*^	.895^*^	.906^*^	.910^*^	1

NB. *SWL* satisfaction with life, *PA* positive affects, *NA* negative affects

* = *p<* 0.001

Additionally, correlations that ranged from moderate to high were established between the factors assessed by the K10 and DASS-21. However, it should be noted that these variations were adequate for the content assessed by the specific and general factors of both instruments.

The coefficients *r* = 0.641 and *r* = 0.762 were observed between the anxiety scores estimated via the K10 and the factors stress and anxiety assessed by DASS-21; *r* = 0.811 between the estimated score of depression on both scales; and *r* = 0.840 between the general scores (psychological distress) estimated via K10 and DASS, respectively. These results support the hypothesis of convergent validity evidence for the Brazilian version of the K10.

## Discussion

The primary objective of this study was the adaptation of the K10 to Brazilian Portuguese, as well as the estimation of different types of validity evidence and reliability (American Educational Research Association [AERA], American Psychological Association [APA],, and National Council of Measurement in Education [NCME], 2014). For this purpose, a comparison was performed between the goodness-of-fit indices of different models used in the literature: the one-dimensional model originally proposed by the ABS (Australian Bureau of Statistics, 2001), a model consisting of two correlated factors (Easton et al., 2017), a model with a second-order factor (Brooks et al., 2006), and the bifactor model. The latter has been hypothesized in the literature based on studies performed with other measures of psychological distress, such as DASS-21 (Peixoto et al: DASS-21: assessment of psychological distress by the Bifactor Model and item analysis, in press). Also, the item properties were evaluated via the IRT graduated response model, as well as validity evidence based on relationships with external variables (satisfaction with life, positive and negative affects, depression, stress, anxiety, and psychological distress).

The results obtained through CFA indicated a goodness-of-fit level significantly higher for the bifactor model consisting of two specific factors (depression and anxiety) and one general factor (psychological distress). These results are consistent with those demonstrated by Brooks et al., 2006. However, the present study also presents evidence that items have higher factorial loads for the general factor at the expense of the specific factors. This fact may corroborate the argument of Sunderland et al. (2012). According to these authors, use of the K10 and the interpretation of its results depend on the objective of the use of the instrument and on the population to be assessed. In community populations and for screening, the study of psychological distress (general factor), at the expense of specific factors (anxiety and depression), may be more compelling. However, in a clinical population, the differentiation of specific problems, assessed via factors found and identified as anxiety and depression, may be more befitting.

As for the interpretation of the factors that make up the internal structure, the strength of the bifactor model in offering more important contributions than second-order models, for example, is worthy of note. According to Reise (2012), in the case of a second-order factor model, the general factor is responsible for explaining the variance of first-order factors, which in turn mirrors the variance of the items. This suggests a certain indistinction in the evaluation of the specific factors. On the other hand, the bifactor model, by indicating a general factor that explains the variance shared among all items, as well as the existence of specific orthogonal multiple factors—each one hypothesized to explain the unique variance of the specific domain (e.g., depression and anxiety)—supports the interpretation and consequently the use of the scores of specific factors and the general factor, simultaneously. Considering that no references were found that explore the structure of the K10 via a bifactor model, these results suggest the present study’s innovative contribution regarding the instrument’s internal structure and helps to advance the discussion that already exists in the international literature regarding the one-dimensionality versus the two-dimensionality of the instrument (e.g., Sunderland et al., 2012). Additionally, the results demonstrate invariance of the measurement model as a function of sex and age groups, which shows the instrument’s potential to be used in future studies that seek to compare levels of distress in these sample strata (Milfont & Fisher, 2010). In this way, as referenced by the literature, it is an adequate instrument for epidemiological studies with population samples with high age dispersion (Furukawa et al., 2003).

Both for the specific factors and for the general factor, good reliability indicators were found (Cronbach’s alpha and McDonald’s omega coefficients equal to 0.891 and 0.887 for the depression factor, 0.886 and 0.886 for the anxiety factor, and 0.923 and 0.922 for the general psychological distress factor, respectively). These indices are considered good and in agreement with the international literature (e.g., Easton et al., 2017; Furukawa et al., 2003; Pereira et al., 2019).

Once the initial validity evidence based on the internal structure and reliability of the Brazilian version of the K10 have been estimated and, consequently, an understanding of the organization of the set of items has been obtained, the application of IRT facilitated an understanding of the adequacy of item properties. The *infit*/*outfit* adjustment indices were all adequate, indicating adequacy of the items to the response pattern expected by the model as a function of the level of psychological distress estimated for the participants (Linacre, 2015).

With regard to the levels of item difficulty, the results provide a better understanding of how the continuum of the psychological distress phenomenon is organized. Thus, item 2 (feeling nervous) and item 5 (feeling anxious and agitated) represent the beginning of the continuum, passing through item 8 (feeling that everything required a great effort) and item 4 (feeling hopeless), representing the middle of the continuum. Finally, item 6 (not being able to keep still) and item 3 (feeling that nothing could calm one) represent the most intense items and are, therefore, at the end of the continuum.

Thus, it may be said that the beginning of the continuum, in other words, a low level of psychological distress, would be characterized by the perception and self-report of agitation and nervousness. As the continuum progresses and with the increase of psychological malaise, this agitation gives way to a lack of energy (e.g., item 8, feeling that everything required a great effort) and a negative view of the world (e.g., item 4, feeling hopeless). At the end of this continuum, characterized as the highest level of distress, there is the perception and self-report of a lack of control over emotions and behavior (e.g., item 3, feeling that nothing can calm one; item 6, not being able to keep still). It may be hypothesized, therefore, that the highest level of distress is associated with a higher perception of difficulty regarding the management and control of emotions. This fact is in agreement with studies in the area that point to greater difficulty in regulating emotions associated with psychiatric disorders (e.g., Mocaiber et al., 2008).

Another important piece of information resulting from the IRT was the association between the levels of item difficulty and levels of intensity in respect of the trait shown by participants on the item map. Results suggest that it is more difficult for the participants to endorse the items where levels of trait intensity are lower than the average. In this regard, the results identify one important strength of the instrument, counting on a higher level of reliability regarding the identification of people with high levels of psychological distress. These characteristics are relevant inasmuch as the K10 represents a screening instrument, with the aim of identifying people with higher levels of psychological distress and who therefore have a greater need for psychological intervention. It is worth mentioning that the items showed positive correlations, like the trait levels estimated for the participants (*theta*), which suggests a good capacity of the participants to recover *theta* levels, even when allocated at points on the scale below the average, for instance.

The present study also assessed the criterion validity of the K10 instrument, comparing it to a similar instrument (DASS-21) and a contrasting one (meaning of life and subjective well-being). The data pointed to significant positive correlations with all the measures similar to the K10, in terms of both its general distress factor and also for the specific factors, anxiety and depression. Indeed, strong correlation can be observed between depression and anxiety in DASS-21 and the K10, as well as between their global measurements (distress). However, in a more detailed analysis, the K10’s anxiety and depression factors have levels of correlation slightly higher with DASS-21’s stress factor than is the case with DASS-21’s anxiety factor. By observing the content of the items of each factor on the DASS-21, it can be seen that the items that comprise the stress factor are related more to psychological suffering while the items relative to the anxiety factor are more related to physical reactions of discomfort (e.g., trembling of the hands) (Lovibond & Lovibond, 1995a, 1995b). In this sense, it may be posited that the K10’s anxiety factor is more directed towards psychological suffering of an anxious kind and, consequently, would be more effective in the detection of anxiety-related psychopathological disorders instead of the physical reactions triggered by anxiety, more characteristic of a crisis of anxiety and not of an anxious disorder or suffering (Dalgalarrondo, 2000). However, future studies should be able to shed light on these interpretations. A study of the instrument’s sensitivity and specificity could help in this regard.

Lastly, it is worth highlighting the benefits of evaluating the psychometric properties of the K10 using classical test theory (CFA, reliability analysis, and relationship with external variables) and modern test theory (RSM). As indicated by the literature (Lin et al., 2019; Nejati et al., 2020), the use of different test theories helps to expand knowledge regarding the psychometric properties of the instrument. Thus, if on one hand classical test methods allowed the estimation of validity evidence based on the internal structure, relationship with external variables and reliability, on the other hand, the modern test methods allowed greater understanding of the properties of the items comprising the K10, better organization of the content of the items in the continuum that represents psychological distress, and the estimation of the characteristics of the respondents independently of the characteristics of the test. Therefore, IRT is a psychometric model that complements the classical model, which allows for new insights in the process of validation and comprehension of the instrument’s strengths and weaknesses in assessing the target construct.

## Conclusion

Although the K10 is a worldwide instrument used to screen for psychological distress, translation, adaptation, validity evidence, and reliability studies regarding the Brazilian population were not yet available in the literature. The present article seeks to fill this gap. Data shows that the K10 have adequate psychometric properties in Brazilian population, presenting evidence of criterion validity and internal structure compatible with those described in the international literature. Also, the invariance of the measurement model regarding sex and age group was observed. Moreover, the indices of reliability are considered high, indicating that the instrument is adequate for measuring what it proposes. Taken altogether, data shows that K10 is a good instrument to assess psychological distress in Brazilian population with potential value to be used in epidemiological studies such as the international ones described in this article.

Despite these advances, the present article did not present studies on sensibility and specificity, or studies with normative data. Neither did the present study evaluate adaptation and validity evidence of the reduced version (K6). Future studies could focus on filling this gap as it is an internationally recognized tool with promising psychometric properties.

## Data Availability

The datasets used and/or analyzed during the current study are available from the corresponding author on reasonable request.
